# Systemic inflammation is an independent predictive marker of clinical outcomes in mucosal squamous cell carcinoma of the head and neck in oropharyngeal and non-oropharyngeal patients

**DOI:** 10.1186/s12885-016-2089-4

**Published:** 2016-02-18

**Authors:** Kellie A. Charles, Benjamin D. W. Harris, Carol R. Haddad, Stephen J. Clarke, Alex Guminski, Mark Stevens, Tristan Dodds, Anthony J. Gill, Michael Back, David Veivers, Thomas Eade

**Affiliations:** School of Medical Sciences (Pharmacology) and Bosch Institute, University of Sydney, Sydney, NSW 2006 Australia; Department of Radiation Oncology, Northern Sydney Cancer Centre, Royal North Shore Hospital, St Leonards, NSW 2065 Australia; Northern Clinical School (Medicine), University of Sydney, Sydney, NSW 2006 Australia; Department of Medical Oncology, Royal North Shore Hospital, St Leonards, NSW 2065 Australia; Department of Anatomical Pathology, Royal North Shore Hospital, St Leonards, NSW 2065 Australia; Cancer Diagnosis and Pathology Research Group, Kolling Institute of Medical Research, University of Sydney, Sydney, NSW 2006 Australia; Department of Surgery, Royal North Shore Hospital, St Leonards, NSW 2065 Australia

**Keywords:** Systemic inflammation, Prognosis, Head and neck cancer, Neutrophil-to-lymphocyte ratio, Overall survival, Recurrence free survival

## Abstract

**Background:**

Currently there are very few biomarkers to identify head and neck squamous cell carcinoma (HNSCC) cancer patients at a greater risk of recurrence and shortened survival. This study aimed to investigate whether a marker of systemic inflammation, the neutrophil-to-lymphocyte ratio (NLR), was predictive of clinical outcomes in a heterogeneous cohort of HNSCC cancer patients.

**Methods:**

We performed a retrospective analysis to identify associations between NLR and clinicopathological features to recurrence free survival (RFS) and overall survival (OS). Univariate analysis was used to identify associations and selected variables were included in multivariable Cox regression analysis to determine predictive value.

**Results:**

A total of 145 patients with stage I-IV HNSCC that had undergone radiotherapy were analysed. Seventy-six of these patients had oropharyngeal cancer and 69 had non-oropharyngeal HNSCC and these populations were analysed separately. NLR was not associated to any clinicopathological variable. On univariate analysis, NLR showed associations with RFS and OS in both sub-populations. Multivariable analysis showed patients with NLR > 5 had shortened OS in both sub-populations but NLR > 5 only predicted RFS in oropharyngeal patients. Poor performance status predicted OS in both sub-populations and current smokers had shortened OS and RFS in non-oropharyngeal patients.

**Conclusions:**

The results show patients with NLR > 5 predict for shorter overall survival. Further prospective validation studies in larger cohorts are required to determine the clinical applicability of NLR for prognostication in HNSCC patients.

**Electronic supplementary material:**

The online version of this article (doi:10.1186/s12885-016-2089-4) contains supplementary material, which is available to authorized users.

## Background

Head and neck squamous cell carcinoma (HNSCC) is an aggressive disease and is the sixth most common cancer worldwide, with approximately 650,000 cases diagnosed worldwide annually and nearly 400, 000 deaths [1, 2]. HNSCC encompasses a wide variety of malignancies deriving from the mucosal epithelium of the upper aerodigestive tract, including lip, oral cavity, paranasal sinuses, nasal cavity, pharynx and larynx [3]. Data from the USA indicates over two-thirds of patients present with advanced-stage disease with either locoregional spread to the lymph nodes or distant metastasis [4]. Historically, up to 50 % of patients will experience locoregional recurrence within 2 years of treatment with limited options for salvage surgery or reirradiation [4, 5]. To date, there is limited molecular characterisation of the driver mutations of the various subtypes of HNSCC, with human papilloma virus (HPV), smoking and alcohol the only identified causative agents. Therefore, understanding the biological mechanisms that lead to cancer progression and identification of prognostic factors are essential to improve the clinical management of HNSCC.

A hallmark of many cancers, including HNSCC, is the presence of a tumour promoting phenotype of chronic, low-grade cancer-related inflammation [6–8]. Recent studies have demonstrated that cancer-related inflammation derives from communication between the host and tumour cells to develop a reciprocal interplay that often results in systemic alterations, immune suppression and evasion and malignant progression [6]. In HNSCC, cancer-related inflammation is characterised by increased circulating concentrations of pro-inflammatory cytokines and acute phase reactant proteins (C-reactive protein, serum amyloid A protein) that enhance the recruitment of circulating neutrophils, monocytes [9], myeloid derived suppressor cells (MDSC) [10, 11], and thus total leucocyte numbers, whilst also inhibiting the recruitment of lymphocytes to the circulation. These changes lead to the development of cancer-related syndromes, including fever, night sweats, fatigue, cachexia and bone and muscle pain [12].

Over the last few years, there has been a proliferation in clinical studies measuring the systemic inflammatory response in cancer patients to identify patients with poor prognosis (reviewed in [7, 13]). One of the key biomarkers of systemic inflammation is the neutrophil-to-lymphocyte ratio (NLR). An NLR score is obtained from a patients full blood count by dividing the absolute neutrophil count by the absolute lymphocyte count. An elevated NLR is strongly related to other inflammatory markers, including the Glasgow Prognostic Score, platelet-lymphocyte ratio and elevated C-reactive protein levels, which have been associated with increased tumour burden and spread of disease. NLR is elevated in patients with laryngeal squamous cell carcinoma compared to patients with benign and precancerous lesions [14]. NLR is also an independent prognostic marker of reduced overall survival (OS) in most epithelial cancers [6, 15].

There have been numerous studies of the prognostic role of NLR in various selected populations of HNSCC. Small studies conducted in site-specific populations of nasopharyngeal, oropharyngeal and oral cavity cancers, showed elevated NLR was predictive of local and regional recurrence or reduced progression free survival and/ or poorer OS [16–20]. Investigations in small cohorts of unselected HNSCC patients have shown that HNSCC patients have an elevated NLR compared to healthy controls and univariate analyses have associated elevated NLR to recurrence, tumour and nodal stage [21–23]. A pilot study in 46 unselected HNSCC patients was conducted by our group and univariate analysis found that NLR was predictive of shorter overall survival [24]. However, in these investigations of heterogeneous populations of HNSCC, multivariable analysis of NLR as prognostic of recurrence free survival (RFS) or OS was not undertaken.

Additionally, literature shows that HPV mediated overexpression of p16 is an important marker of reduced risk for recurrence and survival in HNSCC [25, 26]. Recent in vitro and animal studies of cervical cancer have shown that HPV positive (HPV+) cells are more efficient at producing a pro-inflammatory tumour microenvironment [27] leading to enhanced myeloid cell proliferation in the bone marrow and spleen and increased recruitment of leucocytes to the tumour [28]. Thus, the p16 status of a patient may also alter the inflammatory response and contribute both directly and indirectly to cancer outcomes. Huang et al. [9] identified that p16 positive oropharyngeal cancer patients with high circulating neutrophil levels have a reduced OS and RFS. Interestingly, this association was not seen in the p16 negative oropharyngeal patients. Furthermore, higher levels of circulating lymphocytes were predictive of improved RFS and marginally improved OS in the p16 positive population but not in the p16 negative patients. Additionally, in a study by Ward et al. [29], HPV+ oropharyngeal cancer patients with high or moderate tumour infiltrating lymphocyte expression had significantly improved survival compared to HPV+ low tumour infiltrating lymphocytes and HPV negative (HPV-) patients regardless of lymphocyte expression. This would suggest within the HPV+ oropharyngeal cancer population the systemic and local inflammatory environment may be important for determination of clinical outcomes. In both studies there is a significant minority of HPV+ patients (20 %) that have poor OS. Identification of this high risk group is important in an era of potential treatment de-escalation and introduction of molecularly targeted therapies. In addition, systemic inflammation has not been well investigated as predictive biomarker for all clinical outcomes in the non-oropharyngeal cancer population and identification of the high risk group of patients is also essential.

In this retrospective analysis, we sought to investigate whether NLR was an independent prognostic factor of RFS and OS in a prospectively collected, non-selected HNSCC population from one treatment centre. In addition we investigated whether elevated NLR was associated with clinicopathological features, including p16 status, which may aid in treatment decisions.

## Methods

### Study design

The Northern Sydney Local Health District Human Research Ethics Committee approved this study (1202-056 M). Following local institutional ethical review board approval, we conducted a retrospective analysis of patients with HNSCC treated at the Northern Sydney Cancer Centre between January 2005 and January 2012. Patients were identified using a prospectively collected Head and Neck Cancer Database [30] and informed written consent was obtained from all patients. Eligible patients were required to be 18 years or older, have pathologically confirmed primary mucosal squamous cell carcinoma, undergone radiotherapy based treatment, a minimum follow-up of 12 months (unless deceased) and NLR recorded within 30 days prior to commencing radiotherapy. The patient population included 145 patients with mucosal squamous cell carcinoma of the lip and oral cavity, oropharynx, hypopharynx, nasopharynx or larynx staged I-IV, who had been treated with radiotherapy alone or in combination with surgery and/or chemotherapy.

All patients were initially reviewed at a multidisciplinary head and neck tumour board, which included otolaryngology surgeons, radiation oncologists and medical oncologists who assigned the tumour stage and subsequent management. The patient demographics collected for the present study included age, sex, Eastern Cooperative Oncology Group performance status (ECOG PS), smoking status (current, ex-smoker or non-smoker), primary tumour location, American Joint Committee on Cancer (AJCC; 6th Ed 2002) stage and treatment plan. Additionally, radiotherapy dose, number of fractions and the start and end date of radiotherapy were recorded for each patient. The pre-treatment neutrophil and lymphocyte counts were obtained and the NLR calculated by dividing the neutrophil count by the lymphocyte count. A cut-off of 5 was used to categorise patients with high (NLR > 5) or low (NLR ≤ 5) systemic inflammation. This cut-off was chosen based on the systematic review of the NLR literature in cancer which showed NLR > 5 as a predictive marker of cancer outcomes in over 30 studies of 15,500 cancer patients [7].When available, immunohistochemistry for p16 was performed on formalin fixed paraffin embedded sections using a specific mouse monoclonal antibody (clone JC8, cat SC-56330, Santa Cruz CA, USA) at a dilution of 1 in 10. Staining was interpreted by two observers (TD, AJG) that were blinded to all other clinical and pathological details. Diffuse, strong, full thickness staining was categorised as p16 positive, while absent or focal staining was categorised as p16 negative.

All procedures were in accordance with the ethical standards of the institutional Human Research Ethics Committee on human experimentation and with the Helsinki Declaration of 1975, as revised in 2000.

### Treatment

Patients were treated on a standard department protocols [30] either definitively with radiotherapy (stage I-II), chemoradiotherapy (stage III-IV) or postoperatively in high risk patients. Except for small field larynx treatments, radiotherapy was delivered with sliding window intensity modulated radiation therapy or volumetric modulated arc therapy. For definitive patients treated with chemoradiotherapy, the dose was 70 Gray (Gy) in 35 fractions with weekly cisplatin (40 mg/m^2^) and 63 Gy and 56 Gy respectively to the intermediate and low dose planning target volumes. For patients treated with radiotherapy alone either this fractionation was used or a hypofractionated schedule of 66 Gy in 30 fractions [31]. Postoperative patients received 60 Gy in 30 fractions. Treatment regimens provided to patients remained consistent over the study period.

### Statistical analysis

The primary objective of the study was to determine whether NLR was a predictor of RFS and OS. Patients with oropharyngeal cancer were analysed separately from other tumour sites (lip and oral cavity, nasopharynx, hypopharynx and larynx) due to known difference in disease etiology and patients were assessed for differences between these sub-populations. Additionally, patient demographics were compared between p16 positive and negative oropharyngeal patients. Patient demographics were also assessed for differences in NLR status (NLR ≤ 5 vs NLR > 5) in the total population and the two sub-populations. Statistical tests used for the aforementioned univariate analyses included independent samples *t*-test or Mann Whitney-*U* test for continuous variables and χ2 test or Fisher’s exact test for categorical variables.

Survival outcomes were determined from the start of radiotherapy until the date of the event or death from any cause (date of death obtained from hospital records). The exploratory variables analysed in univariate and multivariable survival analysis were assessed as follows: age (continuous or categorised into 4 groups with equal number of events for univariate survival analysis to assess linear trends), sex (male vs female), ECOG PS (0 vs 1 or 2), smoking (current smokers compared to non-smokers and ex-smokers), AJCC stage (I or II vs III or IV), treatment (chemotherapy used vs other treatments), and NLR (≤5 vs > 5). Patients who had surgery before anti-cancer treatment were not compared to non-surgical patients as surgeries were performed at multiple hospital sites and various types of surgeries were performed depending on the type of HNSCC. Additionally, surgical risk factors were initially included, but due to small numbers subsequently dropped from the analysis. Variables were assessed with Kaplan Meier log rank test and any variable with *p* value < 0.25 was included in a final multivariable Cox regression model to determine significant predictors of RFS and OS with adjustment from other exploratory variables. All data from survival analysis presented as hazard ratios (HR) ± 95 % confidence interval (CI). Statistical tests were two sided with α significance level of 0.05, and *p* values were not adjusted for multiple comparison testing. All analyses performed using IBM SPSS for Windows, Version 20.

## Results

### Patient demographics for total population

A total of 145 patients were included in this retrospective study and patient demographics are detailed in Table 1. This is an expanded dataset that includes 40 patients from a previous pilot study [24]. The median age was 63 years (range, 28–86 years) and the majority of patients were male (79 %) and most had ECOG PS 0 (70 %) or 1 (22 %). Some patients continued smoking through their treatment (26 %) but the majority were ex-smokers (42 %) or non-smokers (30 %). The most common primary disease site was oropharynx (52 %) and the majority of patients had AJCC stage III or IV disease (70 %). Patients were treated with definitive radiotherapy (12 %), postoperative radiotherapy (20 %), definitive chemoradiotherapy (61 %), or postoperative chemoradiotherapy (8 %). Of the 99 patients treated with chemotherapy 89 received weekly cisplatin, 8 received cetuximab and one received carboplatin. Weekly cisplatin was delivered for a median of 6 cycles. One patient did not complete a minimum of 5 cycles of cisplatin and was changed to cetuximab due to toxicity. Radiation treatment was completed without unscheduled breaks in 98 % of patients. Median (range) of neutrophils and lymphocytes was 5.10 (1.10-11.90) and 1.60 (0.20-10.70) x 10^9^ cells/L respectively. And the median (range) of the calculated NLR was 1.60 (0.20-10.70) for the total population. Material for p16 staining was available from 95 of 145 patients (66 %) patients. Systemic inflammation, as determined by elevated NLR > 5, was observed in 20 % of patients. Of the 145 patients in this study, 37 patients (26 %) developed a recurrence or metastasis. At the end of the study, there were 35 deaths and a median 1-year OS of 91 %. Median follow-up time of patients was 29 months (range, 42 days to 7 years).

**Table 1:** Patient demographics

Characteristic	All patients (*N* = 145)^a^	Oropharyngeal (*n* = 76)^a^	Non-oropharyngeal (*n* = 69)^a^	*p* value*
Age, median years (range)	63 (28–86)	59.5 (32–83)	67 (28–86)	<0.01
Sex, *n* (%)				0.5
Male	115 (79)	62 (82)	53 (77)	
Female	30 (21)	14 (18)	16 (23)	
ECOG PS, *n* (%)				<0.001
0	102 (70)	64 (84)	38 (55)	
1	32 (22)	10 (13)	22 (32)	
2	10 (7)	2 (3)	8 (12)	
Missing	1 (1)	-	1 (1)	
Smoking status, *n* (%)				0.2
Non-smoker	44 (30)	24 (32)	20 (29)	
Ex-smoker	61 (42)	36 (47)	25 (36)	
Current smoker	37 (26)	15 (20)	22 (32)	
Missing	3 (2)	1 (1)	2 (3)	
Tumour site, *n* (%)				<0.001
Lip and oral cavity	25 (17)	0 (0)	25 (36)	
Nasopharynx	8 (6)	0 (0)	8 (12)	
Oropharynx	76 (52)	76 (100)	0 (0)	
Hypopharynx	12 (8)	0 (0)	12 (17)	
Larynx	24 (17)	0 (0)	24 (35)	
Tumour stage, *n* (%)				0.05
T1	35 (24)	24 (32)	11 (16)	
T2	52 (36)	29 (38)	23 (33)	
T3	36 (25)	15 (19)	21 (30)	
T4	22 (15)	8 (11)	14 (20)	
Nodal stage, *n* (%)				<0.001
N0	44 (30)	11 (14)	33 (48)	
N1	36 (25)	22 (29)	14 (20)	
N2	60 (41)	41 (54)	19 (28)	
N3	5 (3)	2 (3)	3 (4)	
AJCC stage, *n* (%)				0.2
I	9 (6)	4 (5)	5 (7)	
II	35 (24)	18 (24)	17 (25)	
III	74 (51)	44 (58)	30 (43)	
IV	27 (19)	10 (13)	17 (27)	
p16 tumour status, *n* (%)				<0.001
Negative	48 (51)	7 (16)	41 (80)	
Positive	47 (49)	37 (84)	10 (20)	
Missing	50	32	19	
Treatment, *n* (%)				<0.001
Radiotherapy	17 (12)	10 (13)	7 (10)	
Postoperative radiotherapy	29 (20)	3 (4)	26 (38)	
Chemoradiotherapy	88 (61)	61 (80)	27 (39)	
Postoperative chemoradiotherapy	11 (8)	2 (3)	9 (13)	
Neutrophils, median counts (range) x 10^9^ cells/L	5.10 (1.10 - 11.90)	4.60 (1.10 - 11.90)	5.30 (2.10 - 11.80)	0.2
Lymphocytes, median counts (range) x 10^9^ cells/L	1.60 (0.20 - 10.70)	1.60 (0.40 - 3.40)	1.70 (0.20 - 10.70)	0.1
NLR, median counts (range) x 10^9^ cells/L	3.11 (0.41 - 29.75)	3.11 (1.30 - 29.75)	3.11 (0.41 - 16.00)	0.9
NLR, *n* (%)				0.7
Low (≤5)	116 (80)	60 (79)	56 (81)	
High (>5)	29 (20)	16 (21)	13 (19)	

*Abbreviations*: *ECOG PS* Eastern Cooperative Oncology Group performance status, *AJCC* American Joint Committee on Cancer and *NLR* neutrophil-to-lymphocyte ratio

^*^, appropriate statistical test (Students *t*-test, Mann Whitney-*U*, χ2 test or Fishers exact test) conducted between oropharyngeal and non-oropharyngeal cancer patient excluding missing values and ^a^, missing values indicated in table

### Comparison of demographics between oropharyngeal patients and other primary sites

Table 1 also shows differences between oropharyngeal cancer patients and other primary sites (classified as non-oropharyngeal cancer patients). Patients with oropharyngeal cancer were significantly younger (*p* < 0.01) and had a better ECOG PS (*p* < 0.001). There was a trend that showed oropharyngeal patients had more limited tumours (T1 or T2, 70 % vs 49 %), but more extensive nodal metastases (N2 or N3, 57 % vs 32 %). Therefore, there was no significant difference in final AJCC stage (*p* = 0.2). Oropharyngeal patients rarely had surgery (7 % vs 55 %) and a higher proportion of patients received chemoradiotherapy (82 % vs 52 %). There were no differences in neutrophil and lymphocyte counts in either sub-group. Additionally, systemic inflammation was similar between both populations (NLR > 5, 21 % vs 19 %, *p* = 0.7). Finally, oropharyngeal patients were significantly more likely to show a positive p16 status (84 % vs 20 %, *p* < 0.001).

In the oropharyngeal cancer patients with suitable tissue available for testing, 37 out of 44 tested p16 positive (84 %). This high percentage is consistent with the prevalence of p16 positivity in oropharyngeal patients over the last 5 years at this hospital site (data not shown). Due to the low numbers of p16 negative cases in the oropharyngeal cohort, it was deemed statistically invalid to investigate relationships between NLR and p16 status. Additionally, as the majority of patients were p16 positive there is limited utility for the use of this marker in oropharyngeal populations and, furthermore, there were no significant differences in patient demographics between p16 tested and non-tested oropharyngeal cancer cases (Additional file 1: Table S1). Therefore, in subsequent analysis we combined all oropharyngeal patients and excluded p16 status. In the non-oropharyngeal cancer patients 10 out of 51 tested patients were p16 positive (20 %) with variable rates for each major primary site (lip and oral cavity 4/9 (21 %), nasopharynx 1/1 (50 %), hypopharynx 0/9 (0 %) and larynx 5/21 (24 %)). Due to the lack of consistent evidence for p16 status as a predictive biomarker in non-oropharyngeal cancers and low numbers in each cancer subsite, we have also excluded p16 status from further analysis with clinical outcomes.

### NLR associations with patient demographics and survival

NLR associations to patient demographics in the total population and oropharyngeal and non-oropharyngeal sub-populations are detailed in Table 2. NLR was not associated with age, sex, ECOG PS, smoking status, tumour site, tumour stage, nodal stage, AJCC stage or modality of treatment for any population. Neutrophils, lymphocytes and NLR were significantly associated with NLR status as expected (all *p* values < 0.01).

**Table 2:** Differences in clinical characteristics for high and low NLR groups

Characteristic	All patients (*N* = 145)^a^			Oropharyngeal (*n* = 76)^a^			Non-oropharyngeal (*n* = 69)^a^		
	NLR ≤ 5 (*n* = 116)	NLR > 5 (*n* = 29)	*p* value*	NLR ≤ 5 (*n* = 60)	NLR > 5 (*n* =16)	*p* value*	NLR ≤ 5 (*n* = 56)	NLR > 5 (*n* = 13)	*p* value*
Age, median years (range)	61 (32–86)	67 (28–86)	0.2	57.5 (32–83)	64 (47–81)	0.07	65 (38–86)	70 (28–86)	0.8
Sex, *n* (%)			1			1			1
Male	92 (79)	23 (79)		49 (82)	13 (81)		43 (77)	10 (77)	
Female	24 (21)	6 (21)		11 (18)	3 (19)		13 (23)	3 (23)	
ECOG PS, *n* (%)			0.5			0.5			0.3
0	84 (73)	18 (62)		51 (85)	13 (81)		33 (60)	5 (38)	
1	24 (21)	8 (28)		8 (13)	2 (13)		16 (29)	6 (46)	
2	7 (6)	3 (10)		1 (2)	1 (6)		6 (11)	2 (15)	
Smoking status, *n* (%)			0.8			0.9			0.4
Non-smoker	35 (31)	9 (32)		19 (32)	5 (31)		16 (29)	4 (33)	
Ex-smoker	48 (42)	13 (46)		29 (49)	7 (44)		19 (35)	6 (50)	
Current smoker	31 (27)	6 (21)		11 (19)	4 (25)		20 (36)	2 (17)	
Tumour site, *n* (%)			0.7			-			0.6
Lip and oral cavity	20 (17)	5 (17)		0 (0)	0 (0)		20 (36)	5 (38)	
Nasopharynx	8 (7)	0 (0)		0 (0)	0 (0)		8 (14)	0 (0)	
Oropharynx	60 (52)	16 (55)		60 (100)	16 (100)		0 (0)	0 (0)	
Hypopharynx	9 (8)	3 (10)		0 (0)	0 (0)		9 (16)	3 (23)	
Larynx	19 (16)	5 (17)		0 (0)	0 (0)		19 (34)	5 (38)	
Tumour stage, *n* (%)			0.7			0.3			0.8
T1	31 (27)	4 (14)		22 (37)	2 (13)		9 (16)	2 (15)	
T2	38 (33)	14 (48)		21 (35)	8 (50)		17 (30)	6 (46)	
T3	29 (25)	7 (24)		11 (18)	4 (25)		18 (32)	3 (23)	
T4	18 (16)	4 (14)		6 (10)	2 (13)		12 (21)	2 (15)	
Nodal stage, *n* (%)			0.5			0.8			0.3
N0	37 (32)	7 (24)		10 (17)	1 (6)		27 (48)	6 (46)	
N1	26 (22)	10 (35)		17 (28)	5 (31)		9 (16)	5 (38)	
N2	48 (41)	12 (41)		31 (52)	10 (63)		17 (30)	2 (15)	
N3	5 (4)	0 (0)		2 (3)	0 (0)		3 (5)	0 (0)	
AJCC stage, n (%)			0.9			0.9			0.5
I	7 (6)	2 (7)		4 (7)	0 (0)		3 (5)	2 (15)	
II	27 (23)	8 (28)		14 (23)	4 (25)		13 (23)	4 (31)	
III	59 (51)	15 (52)		34 (57)	10 (63)		25 (45)	5 (38)	
IV	23 (20)	4 (14)		8 (13)	2 (13)		15 (27)	2 (15)	
p16 tumour status, *n* (%)			0.8			0.6			0.2
Negative	39 (51)	9 (47)		5 (14)	2 (25)		34 (85)	7 (64)	
Positive	37 (49)	10 (53)		31 (86)	6 (75)		6 (15)	4 (36)	
Treatment, *n* (%)			0.8			0.8			0.8
Radiotherapy	14 (12)	3 (10)		9 (15)	1 (6)		5 (9)	2 (15)	
Postoperative radiotherapy	24 (21)	5 (17)		3 (5)	0 (0)		21 (38)	5 (38)	
Chemoradiotherapy	68 (59)	20 (69)		46 (77)	15 (94)		22 (39)	5 (38)	
Postoperative chemoradiotherapy	10 (9)	1 (3)		2 (3)	0 (0)		8 (14)	1 (8)	
Neutrophils, median counts (range)	4.55 (1.10-11.80)	6.80 (3.2-11.90)	<0.001	4.40 (1.10-9.30)	7.85 (3.90-11.90)	<0.001	5.15 (2.10-2.80)	6.50 (3.20-11.80)	<0.01
Lymphocytes, median counts (range)	1.75 (0.50-10.70)	1.10 (0.20-1.70)	<0.001	1.65 (0.50-3.40)	1.10 (0.40-1.70)	<0.001	1.90 (0.60-10.70)	1.00 (0.20-1.50)	<0.001
NLR, median counts (range)	2.69 (0.41-5.00)	6.71 (5.09-29.75)	<0.001	2.71 (1.30-4.78)	6.41 (5.09-29.75)	<0.001	2.64 (0.41-5.00)	7.00 (5.55-16.00)	<0.001

*Abbreviations*: *NLR* neutrophil-to-lymphocyte ratio, *ECOG PS* Eastern Cooperative Oncology Group performance status and *AJCC* American Joint Committee on Cancer

*, appropriate statistical test (Students *t*-test, Mann Whitney-*U*, χ2 test or Fishers exact test) conducted between high and low NLR patients and ^a^, missing values excluded from table and statistical analysis

**Fig. 1:**
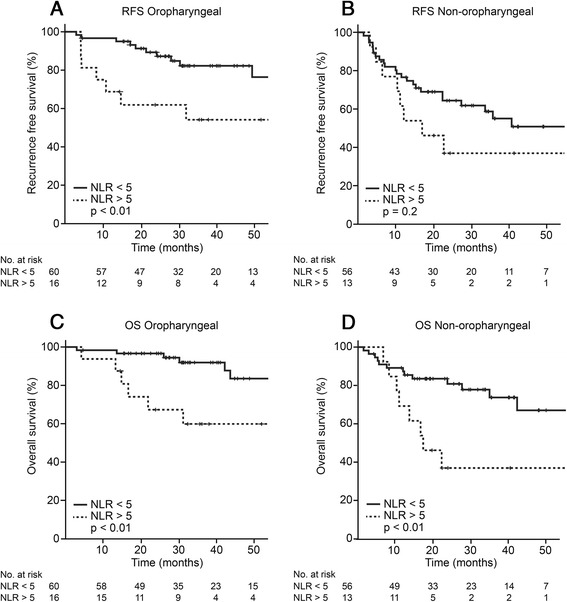
Association of neutrophil-to-lymphocyte ratio to survival outcomes. Neutrophil-to-lymphocyte ratio association to recurrence free survival in oropharyngeal (**a**) and non-oropharyngeal (**b**) patients. Neutrophil-to-lymphocyte ratio association to overall survival in oropharyngeal (**c**) and non-oropharyngeal (**d**) patients. Abbreviations: RFS, recurrence free survival; NLR, neutrophil-to-lymphocyte ratio and OS, overall survival

Univariate survival analysis showed NLR was associated to RFS and OS in the total heterogeneous population, oropharyngeal and non-oropharyngeal subpopulations. In the total population, patients with high NLR had significantly shorter RFS (*p* < 0.01) and OS (*p* < 0.001) and showed a shorter 1-year RFS and OS (62 % vs 87 % and 83 % vs 93 %, respectively). In the oropharyngeal sub-population, high NLR patients also showed a poorer RFS (*p* < 0.01) and OS (*p* < 0.01) with shorter 1-year RFS and OS (60 % vs 97 % and 94 % vs 98 %, respectively, Fig. 1a and c, Table 3). Similarly, non-oropharyngeal patients had a lower RFS (*p* = 0.2) and OS (*p* < 0.01) and shorter 1-year RFS and OS (62 % vs 77 % and 69 % vs 87 %, respectively, Fig. 1b, and d, Table 3).

**Table 3:** Univariate and multivariable analysis of OS and RFS in oropharyngeal and non-oropharyngeal patients

	Overall survival				Recurrence free survival			
Variable	Univariate, HR (95 % CI)	*p* value*	Multivariable, HR (95 % CI)	*p* value**	Univariate, HR (95 % CI)	*p* value*	Multivariable, HR (95 % CI)	*p* value**
Oropharyngeal patients (*n* = 76)^a^								
Age (continuous)	1.07 (1.01-1.12)	0.03	1.03 (0.97-1.10)	0.3	1.05 (1.00-1.10)	0.08	1.02 (0.97-1.08)	0.4
Sex (males vs females)	*No females died*				*No females had recurrence*			
ECOG PS (0 vs 1–2)	4.08 (1.38-12.12)	<0.01	4.36 (1.18-16.06)	0.03	3.33 (1.24-8.89)	0.01	2.92 (0.95-8.97)	0.07
Smoking status		<0.01		0.2		0.03		0.3
(current smoker^b^ vs non-smoker)	0.17 (0.04-0.70)		0.34 (0.07-1.64)		0.31 (0.01-0.98)		0.53 (0.15-1.88)	
(current smoker^b^ vs ex-smoker)	0.22 (0.07-0.80)		0.28 (0.07-1.09)		0.28 (0.09-0.85)		0.40 (0.12-1.31)	
AJCC stage (I-II vs III-IV)	0.78 (0.26-2.34)	0.7	-		0.82 (0.31-2.19)	0.7	-	
Treatment (CRT and CRT + surgery vs RT and RT + surgery)	0.50 (0.46-4.93)	0.5	-		0.63 (0.32-4.00)	0.6	-	
NLR (≤5 vs > 5)	4.96 (1.66-14.80)	<0.01	4.60 (1.26-16.80)	0.02	3.50 (1.38-8.90)	<0.01	3.01 (1.07-8.45)	0.04
Non-oropharyngeal patients (*n* = 69)^c^								
Age (continuous)	1.02 (0.99-1.06)	0.8	-		1.01 (0.98-1.032)	0.9	-	
Sex (males vs females)	1.05 (0.38-2.87)	0.9	*-*		0.81 (0.33-1.96)	0.6	-	
ECOG PS (0 vs 1–2)	3.37 (1.36-8.37)	<0.01	2.57 (0.98-6.76)	0.04	1.66 (0.82-3.36)	0.2	1.49 (0.70-3.21)	0.2
Smoking status		0.04		<0.001		0.02		<0.001
(current smoker^b^ vs non-smoker)	0.18 (0.04-0.79)		0.16 (0.03-0.76)		0.35 (0.14-0.87)		0.35 (0.14-0.90)	
(current smoker^b^ vs ex-smoker)	0.56 (0.22-1.44)		0.34 (0.12-0.94)		0.38 (0.16-0.90)		0.32 (0.13-0.79)	
AJCC stage (I-II vs III-IV)	1.43 (0.56-3.70)	0.5	-		1.53 (0.68-3.42)	0.3	-	
Treatment (CRT and CRT + surgery vs RT and RT + surgery)	0.85 (0.46-2.57)	0.8	-		1.01 (0.50-2.05)	1	-	
NLR (≤5 vs > 5)	3.32 (1.36-8.10)	<0.01	3.64 (1.34-9.87)	0.02	1.76 (0.79-3.96)	0.2	2.02 (0.83-4.91)	0.1

*Abbreviations*: *HR* hazard ratio, *ECOG PS* Eastern Cooperative Oncology Group performance status, *AJCC* American Joint Committee on Cancer, *CRT* chemoradiotherapy, *RT* radiotherapy and *NLR* neutrophil-to-lymphocyte ratio

*, *p* value from Kaplan-Meier logrank test; **, *p* value from Cox regression log likelihood ratio test; ^a^, one patient missing smoking status; ^b^, referent group; and ^c^, missing 3 patients (two patients missing smoking status and one patient missing ECOG status).

### Predictors of recurrence free survival and overall survival

Univariate survival analysis results for oropharyngeal and non-oropharyngeal populations are detailed in Table 3. These analyses showed that ECOG PS, smoking status and NLR associated to RFS and OS in both populations and were included in final Cox regression models as p values were all less than 0.25. Additionally, age was associated with OS and RFS but only in the oropharyngeal population. Variables not associated with any survival outcome from univariate analysis included sex, AJCC stage and treatment modality. Sex was not analysed in oropharyngeal sub-population as no females had recurrence or died in the study period.

Multivariable analysis results are described in Table 3. In oropharyngeal patients, age was no longer associated with RFS or OS once adjusted for by other variables. However, patients with poorer ECOG PS (1 or 2) had a significantly increased hazard of death (4.4 (1.2-16.1), *p* = 0.03) and a trend for increased hazard of recurrence (2.9 (1.0-9.0), *p* = 0.07). Smoking status was not significantly predictive of OS and RFS in oropharyngeal patients. A high systemic inflammation status, NLR > 5, was significantly associated to increased hazard of death (4.6 (1.3-16.8), *p* = 0.02) and recurrence (3.0 (1.1-8.5), *p* = 0.04) in this sub-population.

In non-oropharyngeal patients, poor ECOG PS showed an increased hazard of death (2.6 (1.0-6.8), *p* = 0.04) but no association was seen for RFS. Non-oropharyngeal patients who continued to smoke through treatment had significantly increased hazard, compared to non-smokers and ex-smokers, for recurrence and death (both *p* values < 0.001). An NLR > 5 was significantly associated with increased hazard of death (3.7 (1.3-9.9), *p* = 0.02) but no strong association to RFS was seen.

## Discussion

NLR is an easily obtainable, inexpensive marker of systemic inflammation that may assist in clinical decisions regarding recurrence and survival in a heterogeneous HNSCC population. This study aimed to investigate the predictive role of NLR in an unselected population of HNSCC patient, but we found that oropharyngeal patients had significant differences in baseline characteristics compared to non-oropharyngeal patients. As expected from the growing literature, a very high percentage of oropharyngeal patients were p16 positive (84 %). Thus, we conducted total and sub-site analyses due to the differences reflecting the potential diverging molecular etiology of oropharyngeal and non-oropharyngeal disease. In univariate analysis, NLR status did not associate with any other clinicopathological variables other than neutrophil and lymphocyte levels in either subgroup as expected. Patients with an elevated NLR were associated with shorter RFS and OS in both oropharyngeal and non-oropharyngeal populations. Univariate survival analysis showed ECOG PS, smoking status, age and NLR associated with RFS and OS to varying degrees in both populations. Multivariable analysis confirmed NLR significantly predicted RFS in oropharyngeal patients only, while NLR strongly predicted OS in both sub-populations. Additionally, ECOG PS significantly showed associations to OS in oropharyngeal patients and non-oropharyngeal patients. Interestingly, smoking status remained predictive of RFS and OS only in non-oropharyngeal patients. This may not be unexpected considering the low numbers of p16 negative patients in the oropharyngeal cancer cohort, which may represent the contribution of smoking habits in the causation of disease in these patients.

The majority of oropharyngeal patients were p16 positive in this study and oropharyngeal patients were younger, had better ECOG PS but had increased nodal spread compared to non-oropharyngeal patients. Eighty-four percent of tested oropharyngeal patients had p16 positive tumours. This percentage is comparable to other American, Swedish and British studies (summarised in [32]) although higher than the average rate (~40 %) in most developed countries. Patients with p16 positive tumours are generally younger [33] and have been noted to have better ECOG PS and higher nodal stages when compared to p16 negative patients [34, 35]. The high prevalence of p16 in the oropharyngeal population most likely accounts for the younger age and better ECOG PS compared to non-oropharyngeal patients seen in this study. The higher nodal stage but improved outcomes in p16 positive patients is the most likely cause of AJCC stage not being significant in our study, similar to other reports [34]. The 3-year OS of oropharyngeal patients was 86 % and 69 % for p16 positive and negative patients respectively, which is comparable to larger studies [36–38]. In vitro studies with p16 positive HNSCC cells lines have shown that these cells are more radiosensitive [39]. Oropharyngeal patients in our unit are unlikely to undergo primary surgical intervention due to the perceived high risk of morbidity if extensive surgery is required. Our excellent rates of locoregional control in this population further support this recommendation. However with availability of transoral robotic assisted surgery [40], a biomarker to predict a poor performing oropharyngeal subgroup may aid selection of patients for surgery in the future.

With decreasing smoking rates due to extensive anti-smoking campaigns, as seen in countries such as Australia, HPV+ oropharyngeal cancer is increasingly becoming the prominent subtype. Therefore, additional predictive biomarkers of clinical outcomes are needed within the HPV+ or p16 positive oropharyngeal cancer population. Additionally, classification of patients as HPV+ is not without difficultly as the various techniques of assessment produce variable results and there is no universally agreed classification system. The results of this study show that on a background of high p16 positive status, elevated NLR was associated with recurrence and survival outcomes under univariate analysis and many of the recurrences and deaths occurred within the first year following radiotherapy. Multivariable analysis showed that NLR remained a predictor of OS independent of AJCC stage, tumour site, treatment modality and sex in oropharyngeal and non-oropharyngeal sub-populations. Additionally, NLR also predicted RFS in oropharyngeal patients. The results of this study identified NLR as a prognostic marker of OS in an unselected HNSCC cohort, supporting previous findings from other studies in nasopharyngeal, oral squamous cell carcinoma and preliminary investigations in unselected HNSCC cohorts [14, 16–22, 41]. These findings are also consistent with other cancer types including other head and neck associated cancers, such as thyroid cancer [42, 43].

The association between NLR and poor OS and recurrence is not well understood. However, it is hypothesised that elevated NLR reflects a more aggressive tumour phenotype that is immune evasive and/or suppressive. Elevated NLR is more often seen in patients with advanced disease, as denoted by increased AJCC stage, tumour depth of invasion or metastatic spread [7]. In our study, we did not find evidence to confirm NLR was associated with higher AJCC staging and thus may represent aspects reflecting immune suppression. Recent analysis conducted by The Cancer Genome Atlas project, shows that within the HPV+ population of HNSCC there is an increase in loss of *TNF receptor-associated factor 3* gene and presence of activating mutations in *PIK3CA* gene, which enhance NF-κB signalling and promote a pro-inflammatory microenvironment [44]. This data supports the role of cancer-related inflammation in determining the outcomes of HPV+ HNSCC patients.

In the tumour microenvironment, innate immune cells, such as neutrophils, macrophages and myeloid derived suppressor cells, regulate both immune surveillance and suppression [45]. Increased abundance of these cells is observed in more advanced stages of HNSCC and is associated with poorer survival [9–11, 46]. Mechanistic studies conducted in animal models and ex vivo cultures of immune cells from HNSCC patients have demonstrated that myeloid derived suppressor cells are critical for regulating the immunosuppressive phenotype and function of co-operating lymphoid-derived cells in the tumour and circulation [10, 11, 47, 48]. In terms of adaptive immune cells, the low infiltration of T cells, particularly T regulatory cells, combined with functional deficits in T helper cells, cytotoxic T cells and natural killer cells leads to the highly immune suppressive tumour microenvironment that allows for unrestrained tumour growth [29, 49–52].

Improved understanding of the various interactions of the tumour and immune system suggest that the ideal biomarker would measure both the innate and adaptive immune response, such as the NLR, as this may provide a better indication of the impact of tumour growth on both arms of the host immune response. In a mixed cancer population (not including HNSCC patients) elevated NLR was found to positively correlate with circulating MDSC levels and suppression of lymphocyte function [53]. However, there is no evidence to date that specifically links elevated NLR to immune cell behaviour in HNSCC tumours or circulation. Unfortunately, we do not have blood samples from our patient cohort, but it would be interesting to investigate circulating NLR values in studies that have measured peripheral blood and tumoural MDSC or T cell populations and overall survival to clarify the biological relationships between NLR and immune suppression in cancer.

More recently, the NLR has been suggested as a Phase I clinical trial patient selection tool by the Royal Marsden Hospital, UK [54]. Pharmacological inhibitors of key immunosuppressive mediators (anti-PD1 or PD ligand 1 antibodies, STAT3 and PDE5 inhibitors) have been shown to reduce the number and function of MDSC, Tregs and/or immune T cell-mediated anti-tumour responses in mice and are increasingly being investigated in clinical trials [11, 55, 56] . New data from the The Cancer Genome Atlas [44] has also suggested novel pathways for intervention, such as the PIK3 pathway due to activating mutations in *PI3KCA* for HPV+ cancers. Thus, NLR could be useful as inclusion criteria for clinical trial participation investigating these molecular targeted and immune modulating therapies.

In addition to NLR predicting survival outcomes, other exploratory variables including smoking status and ECOG PS were predictive of RFS and OS. Smoking status was a significant predictor of RFS and OS but only in the non-oropharyngeal population. Smoking is not only a risk factor for the development of head and neck cancer but patients who maintain smoking during treatment are also at increased hazard of worse clinical outcomes [57]. Patients with poorer ECOG PS had an increased hazard of death in both sub-populations which has been suggested previously in HNSCC [58] and observed in other advanced cancers [59].

This study is limited by inherent selection bias due to the retrospective analysis of this study and being conducted in one metropolitan area hospital. However, this cohort reflects the heterogeneous nature of HNSCC in the community. Our population has a large proportion of p16 positive oropharyngeal tumours with comparable clinical outcomes to other international sites. Similar to other cancers (breast, colon, lung), the management of this patient group will change in the future from one single treatment to individualised treatments based on patient and tumour characteristics. One of the main limitations of the study was the incomplete analysis of p16 status in all patients. It would be of interest to investigate if the p16 positive oropharyngeal patients alone mimic the results of the total oropharyngeal population. Due to the high rates of positive patients it is probable that the results would be similar, unfortunately, our study did not have large enough numbers of tested patients to confirm this assumption. Although all patients had radiotherapy and chemotherapy at the one site, surgery was conducted over multiple hospital sites. Unfortunately, we were unable to collect diagnostic blocks from some surgical sites and private pathology laboratories. As such, we assumed based on the lack of significant differences in major covariates in the tested and untested populations and consistency with overall rates of p16 positive oropharyngeal cancer patients in our local area health service, that p16 status was not a statistically relevant covariate in our patient population. Using this assumption we may have missed an important interaction between p16 and NLR. In addition, we used p16 immunohistochemistry as the method for HPV positivity. There is a known discordance between DNA and protein detection methods [60, 61]. The p16 positive immunohistochemistry staining method, as performed in this paper, assumes that the overexpression of p16 is predominantly due to HPV infections, however HPV-independent mechanisms such as alterations in the retinoblastoma pathway may also drive p16 expression [44]. A variety of DNA-based and immunohistochemical methods have been used in various studies and consensus methods are being developed.

## Conclusions

We have conducted an extensive analysis of clinicopathological variables and identified that NLR, ECOG PS and smoking status are predictive of OS and RFS in sub-populations of a heterogeneous HNSCC population. NLR is an inexpensive, routinely available blood test based marker that would be a valuable tool for use in clinical decision-making. The association of NLR to RFS and OS is believed to relate to potential roles of inflammation in regulating cancer progression and immune evasion. Thus, NLR may help identify patients at high risk of recurrence and early death and indicates that this subset of patients may require additional treatments in order to improve their prognostic outlook. Additionally, the NLR has potential utility in selecting patient populations in clinical trials using immune modulating therapies. Further larger prospective studies are required in HNSCC populations to improve the clinical outcomes of all patients.

## Additional file

Additional file 1: Table S1. Patient demographic differences between p16 tested and non-tested oropharyngeal patients. (XLSX 11 kb)

## Abbreviations

AJCC: American Joint Committee on Cancer
CI: Confidence interval
ECOG PS: Eastern Cooperative Oncology Group performance status
FDG-PET: Fluorodeoxyglucose - positron emission tomography
Gy: Gray
HNSCC: Head and neck squamous cell cancer
HPV: Human papilloma virus
HR: Hazard ratio
MDSC: Myeloid derived suppressor cells
NLR: Neutrophil-to-lymphocyte ratio
OS: Overall survival
RFS: Recurrence free survival
